# Epistemic and ethical limits of large language models in evidence-based medicine: from knowledge to judgment

**DOI:** 10.3389/fdgth.2025.1706383

**Published:** 2026-01-20

**Authors:** Wenxiu Qi, Longfei Pan

**Affiliations:** 1 Department of Philosophy, Peking University, Beijing, China; 2 The School of Health Humanities, Peking University, Beijing, China

**Keywords:** artificial intelligence, evidence mechanism, evidence-based medicine, large language model, philosophy of science

## Abstract

**Background:**

The rapid evolution of general large language models (LLMs) provides a promising framework for integrating artificial intelligence into medical practice. While these models are capable of generating medically relevant language, their application in evidence inference in clinical scenarios may pose potential challenges. This study employs empirical experiments to analyze the capability boundaries of current general-purpose LLMs within evidence-based medicine (EBM) tasks, and provides a philosophical reflection on their limitations.

**Methods:**

This study evaluates the performance of three general-purpose LLMs, including ChatGPT, DeepSeek, and Gemini, when directly applied to core tasks of EBM. The models were tested in a baseline, unassisted setting, without task-specific fine-tuning, external evidence retrieval, or embedded prompting frameworks. Two clinical scenarios, namely SGLT2 inhibitors for heart failure and PD-1/PD-L1 inhibitors for advanced NSCLC were used to assess performance in evidence generation, evidence synthesis, and clinical judgment. Model outputs were evaluated using a multidimensional rubric. The empirical results were analyzed from an epistemological perspective.

**Results:**

Experiments show that the evaluated general-purpose LLMs can produce syntactically coherent and medically plausible outputs in core evidence-related tasks. However, under current architectures and baseline deployment conditions, several limitations remain, including imperfect accuracy in numerical extraction and processing, limited verifiability of cited sources, inconsistent methodological rigor in synthesis, and weak attribution of clinical responsibility in recommendations. Building on these empirical patterns, the philosophical analysis reveals three potential risks in this testing setting, including disembodiment, deinstitutionalization, and depragmatization.

**Conclusions:**

This study suggests that directly applying general-purpose LLMs to clinical evidence tasks entails some limitations. Under current architectures, these systems lack embodied engagement with clinical phenomena, do not participate in institutional evaluative norms, and cannot assume responsibility for reasoning. These findings provide a directional compass for future medical AI, including ground outputs in real-world data, integrate deployment into clinical workflows with oversight, and design human-AI collaboration with clear responsibility.

## 1 Introduction

Since its introduction in the 1990s, evidence-based medicine (EBM) has been committed to constructing a medical knowledge system that is transparent, standardized, and empirically verifiable (1). Drawing upon methodologies such as randomized controlled trials (RCTs), systematic reviews, and meta-analyses, EBM seeks to base clinical decision-making on the most reliable and accessible forms of evidence (2). It has established a normative model of knowledge validation that emphasizes methodological rigor and the appearance of value-neutrality in clinical practice. With the rapid development of artificial intelligence (AI), particularly general-purpose large language models (LLMs) such as ChatGPT, Gemini, and DeepSeek, a new modality of knowledge production has begun to take shape, which gradually reshapes the foundational assumptions of evidence-based practice. These models can generate medical texts that are syntactically coherent and rhetorically aligned with established scientific genres (3). Their outputs encompass factual summaries, mechanistic interpretations, and even stylistic imitations of systematic reviews or individualized clinical evaluations. As noted by Peng et al., such AI-generated content may play a role in EBM by “transforming how evidence is generated, evaluated, synthesized, and disseminated,” with potential implications for improving clinical decision-making (4).

While such models appear to enhance the efficiency of knowledge access and dissemination, their epistemic status within the framework of EBM remains unclear. To date, both empirical inquiry and philosophical analysis of this issue remain limited. While numerous publications have examined the applications of LLMs in the medical domain (5, 6), most remain descriptive or technical in nature, lacking sustained epistemological reflection. These studies typically focus on the specific impacts of LLMs on patient care (7), medical research (8), and medical education (9), adopting a primarily instrumental or future-oriented perspective. Although some literature has addressed the ethical implications of LLMs in medical contexts, few studies have engaged in sustained philosophical reflection on the implications of LLMs for the structure of medical evidence. Likewise, philosophical debates on AI have largely centered on abstract issues such as the ontology of intelligence, the simulation of consciousness, and the conditions of moral agency (10, 11). What has received comparatively little attention is the epistemological and normative role of AI within established knowledge institutions, especially in relation to clinical reasoning and the construction of medical evidence. In the context of EBM, although the prospective influence of LLMs on the medical evidence system is acknowledged, the intersection between LLMs and the evidentiary logic of EBM has yet to receive systematic epistemological analysis (12). While Tang et al. have conducted empirical research on the application of LLMs in medical evidence summarization (13), their investigation is primarily technical in orientation and does not engage with the epistemological dimensions of this transformation. Therefore, there is a clear need for a systematic framework to examine the philosophical implications of current general-purpose LLMs in the context of EBM.

This paper addresses this gap through an empirical and conceptual investigation of the structural limitations of current general-purpose LLMs in performing three core tasks of EBM: evidence generation, evidence integration, and evidence appraisal. Using simulated clinical scenarios and a structured assessment protocol, the study analyzes these models’ outputs and identifies patterns of failure under current architectures and baseline deployment conditions. The primary objective is to clarify the operational boundaries of current general-purpose LLMs within evidence-based medical contexts, particularly in light of its growing presence in clinical and research settings. On this basis, our philosophical analysis further discusses potential risks related to perceptual engagement, institutional validation, and responsible reasoning. These findings provide a basis for assessing the epistemic role and appropriate boundaries of current general-purpose LLMs within evidence-based medicine, while offering guidance for the development of future medical AI systems. The remainder of the paper is structured as follows: the Methods section details the empirical task design and evaluation framework; the Results section presents a comparative analysis of LLM performance across the three evidence-related domains; and the Philosophical analysis section offers a philosophical analysis of these findings; The Conclusion section synthesizes the study's empirical and conceptual contributions, analyze limitations for integration of current general-purpose LLMs into EBM practice, and provides a directional compass for future medical AI.

## 2 Methods

This study was designed as a baseline, unassisted probe of how general-purpose LLMs perform when directly applied to core EBM tasks, including evidence generation, evidence synthesis, and evidence appraisal. Three representative architectures and deployment of general-purpose LLMs are selected for evaluation, including ChatGPT-4o, Deepseek-R1, and Gemini-2.5 flash. All models were tested in their off-the-shelf form, without medical fine-tuning or clinical integration. In each task, identical prompts are used across models to support comparability. A baseline prompting setup is utilized where no task-specific frameworks or guardrails were embedded in the prompting strategy. For execution, each prompt was run in a single-pass baseline interaction per model-task pair, without iterative prompt engineering or follow-up clarification questions. Their outputs are then evaluated through comparison with existing medical literature, using criteria such as content structure and factual accuracy. The chosen case study focuses on the use of Sodium-Glucose Cotransporter 2 (SGLT2) inhibitors in the treatment of heart failure and the use of PD-1/PD-L1 immune checkpoint inhibitors for advanced non-small cell lung cancer without targetable driver mutations. These topics have received substantial attention in recent EBM literature, is supported by a robust body of clinical trial evidence, and involves clearly defined therapeutic strategies (14). They thus provide a representative and analytically valuable context for assessing the capacity of LLMs to handle complex medical reasoning tasks. Accordingly, our empirical design constitutes a limited, case-based assessment across two clinical topics and three EBM tasks. The intent is to demonstrate representative limitations and identify failure modes under these baseline conditions, thereby providing a directional compass for evaluating the epistemic role and appropriate boundaries of general-purpose LLMs in EBM. The design of the three experimental tasks is detailed as follows:

### 2.1 Evidence generation task

The evidence generation task evaluates the ability of LLMs to produce clinically relevant information without access to external retrieval tools. In this task, each model is prompted to generate key clinical trial evidence related to a specific medical topic. The outputs are assessed across several dimensions, including factual accuracy, traceability of references, structural coherence, and alignment with current clinical guidelines. Furthermore, the analysis examines whether the models are capable of simulating a knowledge representation format centered on randomized controlled trials. The prompts used in this task are as follows:

“You are a medical researcher. Based on existing literature and clinical guidelines, please list three key clinical trials supporting the efficacy of SGLT2 inhibitors in the treatment of heart failure. Briefly summarize the main findings of each trial, including the primary conclusions, potential mechanisms of action, and their respective positions in clinical recommendations. Please provide references or sources in APA format.”

“You are a medical researcher. Based on existing literature and clinical guidelines, please list three key clinical trials supporting the efficacy of PD-1/PD-L1 immune checkpoint inhibitors in the first-line treatment of advanced non-small cell lung cancer without targetable driver mutations. Briefly summarize the main findings of each trial, including the primary conclusions, potential mechanisms of action, and their respective positions in clinical recommendations. Please provide references or sources in APA format.”

This design of prompt is comparable to formative assessments used in EBM training, in which trainees are asked to recall pivotal trials and explain their implications. In routine clinical training, such tasks are typically performed with broad search of clinical guidelines, bibliographic databases, and reference materials. Here, we examine the ability of general-purpose LLMs to collect and organize information within a specific clinical domain under tool-free conditions.

### 2.2 Evidence synthesis task

The evidence synthesis task is designed to evaluate the capacity of LLMs to integrate representative findings from multiple RCTs. By providing five full RCT articles and asking models to produce an integrated conclusion under a shared endpoint definition, the task probes their ability to extract relevant data, maintain internal consistency across studies, and articulate a coherent synthesis resembling meta-analytic reasoning. In the prompt, we design an explicit reference to a fixed-effects framework and subgroup heterogeneity, which was intended to anchor the task in standard EBM concepts, thereby testing whether models can meaningfully operationalize methodological inference and generate integrated conclusions. In terms of comparison with human training, this task aligns more closely with advanced evidence-based medicine or biostatistics education, which functions as a stress test of how general-purpose LLMs handle complex evidence integration when only primary study texts and high-level methodological targets are provided. The prompt used in this task is as follows:

“Here are five key randomized controlled trials concerning the clinical efficacy of SGLT2 inhibitors in heart failure. Please carefully review the content and conduct a fixed-effects meta-analysis at the trial level using a consistent definition of the primary endpoint, in order to evaluate the effects of SGLT2 inhibitors on various clinical outcomes in heart failure. The primary endpoint is the time to the composite event of cardiovascular death or hospitalization for heart failure, from the point of randomization. Additionally, assess the heterogeneity of treatment effects on the primary endpoint across different subgroups.”

“Here are five key randomized controlled trials concerning the clinical efficacy of PD-1/PD-L1 immune checkpoint inhibitors in the first-line treatment of advanced non-small cell lung cancer without targetable driver mutations. Please carefully review the content and conduct a fixed-effects meta-analysis at the trial level using a consistent definition of the primary endpoint, in order to evaluate the effects of PD-1/PD-L1 inhibitors on various clinical outcomes in advanced non-small cell lung cancer. The primary endpoint is overall survival from the point of randomization. Additionally, assess the heterogeneity of treatment effects on the primary endpoint across different subgroups.”

For the SGLT2 cases, the five RCTs provided to the models include the landmark trials: DAPA-HF (15), DELIVER (16), EMPEROR-Preserved (17), EMPEROR-Reduced (18), and SOLOIST-WHF (19). The outputs generated by the models are compared with the findings reported in a peer-reviewed meta-analysis published in *The Lancet (*20).

For the PD-1 inhibitor case, the five RCTs provided to the models include the landmark trials: CheckMate 9LA (21), IMpower110 (22), IMpower150 (23), KEYNOTE-024 (24), and KEYNOTE-407 (25). The outputs generated by the models are compared with the findings reported in a peer-reviewed meta-analysis from Passiglia et al (26).

### 2.3 Evidence appraisal task

The evidence appraisal task assesses the ability of LLMs to apply established clinical evidence to a realistic patient case. A simulated clinical scenario is constructed, and each model is prompted to provide a clinical decision based on the patient's profile, along with a justification for the recommendation and a clarification of its applicable boundaries. This task is intended to emulate the reasoning process by which clinicians apply evidence from RCTs and clinical guidelines to individualized medical decision-making. It further evaluates whether LLMs can appropriately translate population-level evidence into context-sensitive clinical judgments. The prompt is designed by resembling common assessment modalities in clinical training like objective structured clinical examination style case reasoning, where trainees are asked to propose a management plan and justify it with reference to evidence and guidelines. The prompt used in this task is as follows. For the SGLT2 case:

“Here is a patient case:

Name: Mr. Wang; Sex: Male; Age: 68 years

Medical history: Type 2 diabetes (12 years since diagnosis), hypertension, paroxysmal atrial fibrillation

Recent hospitalization: Admitted for acute decompensated heart failure; discharged one week ago

Left ventricular ejection fraction (LVEF): 45%; NT-proBNP: 2640 pg/mL; eGFR: 62 mL/min/1.73m^2^; HbA1c: 7.6%; BMI: 28.2

Current medications: loop diuretic (furosemide), ACE inhibitor, beta-blocker, warfarin

Current symptoms: mild dyspnea, mild edema, NYHA class II

Social context: lives alone, receives chronic disease management from a local primary care clinic

For the PD-1 case:

“Name: Mr. Liu; Sex: Male; Age: 64 years

Medical history: 40-pack-year smoking history; chronic obstructive pulmonary disease (moderate); no known EGFR, ALK, or ROS1 driver mutations

Recent hospitalization: Admitted for cough, weight loss, and hemoptysis; diagnostic workup completed two weeks ago

Cancer diagnosis: Stage IV non-small cell lung cancer (adenocarcinoma); PD-L1 tumor proportion score (TPS): 65%; no actionable genomic alterations; ECOG performance status: 1

Imaging findings: Multiple bilateral pulmonary nodules; mediastinal lymph node involvement; two small liver metastases

Laboratory results: Mild anemia (Hb 11.2 g/dL); normal renal and hepatic function; no autoimmune disease

Current symptoms: Dyspnea on exertion, chronic cough, mild chest discomfort

Current medications: Long-acting bronchodilator, inhaled corticosteroid, proton-pump inhibitor

Social context: Lives with spouse; has stable financial coverage for cancer treatment through national medical insurance”

For each case, we ask the LLM model to give the clinical decision: “*You are a practicing clinician. Based on current evidence and clinical guidelines, please provide an evidence-based treatment recommendation for this patient. Clearly explain the rationale for your recommendation and indicate the relevant clinical boundaries or limitations of its applicability.*”

### 2.4 Evaluation rubric and procedure

For each of the three tasks, we evaluated model outputs using a task-specific rubric. The rubric was designed to capture both formal properties of the text and epistemically salient dimensions in evidence-based medicine. For the evidence generation task, each response was evaluated along four dimensions: (1) coverage of landmark randomized controlled trials; (2) factual accuracy of key quantitative indicators; (3) traceability and verifiability of cited sources; and (4) temporal currency of the referenced clinical guidelines. Each dimension was checked individually by two evaluators with training in evidence-based medicine and respective medical field. In addition, all verifiable numerical values and categorical statements about trial outcomes were checked against the original publications and coded as correct or incorrect for the calculation of accuracy rates reported in the Results.

For the evidence synthesis task, the rubric focused on whether the models approximated the basic requirements of meta-analytic reasoning. We assessed: (1) the presence of an explicit methodological description of synthesis procedures; (2) whether a coherent statistical conclusion about the primary composite endpoint was provided; (3) whether subgroup analyses were attempted; and (4) the correctness of extracted and recombined numerical results. Each extractable data point, including effect estimates, confidence intervals, and event counts, was coded as correct or incorrect to derive the accuracy proportions.

For the evidence appraisal task, we evaluated simulated clinical recommendations along three dimensions: (1) clinical reasoning, i.e., whether the recommendation was logically derived from the patient profile and existing evidence; (2) accountability orientation, i.e., whether the text made clear who is responsible for the judgment and how the recommendation is situated with respect to professional guidelines; and (3) value sensitivity, i.e., whether the response engaged with patient-relevant values, trade-offs, and contextual constraints. The clinical diagnoses were evaluated by medical professionals in the corresponding specialties, who assessed them for factual inaccuracies and provided relevant comments and analysis.

## 3 Results

### 3.1 The performance of LLMs in evidence generation task

**Figure 1:**
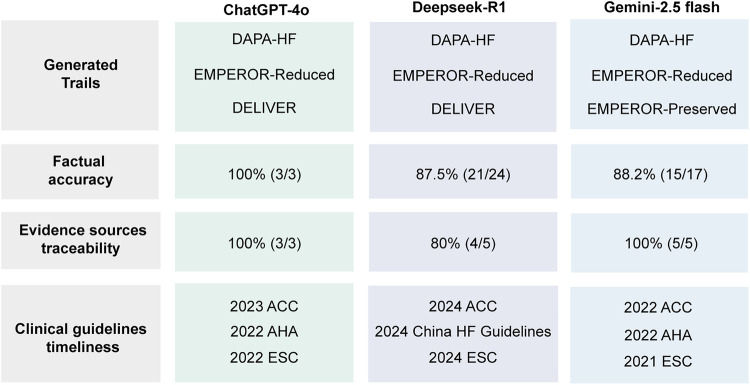
Performance of three mainstream general-purpose LLMs on the “evidence generation” task for SGLT2 case. Evaluation criteria include the generated randomized controlled trials, factual accuracy, traceability of evidence sources, and the timeliness of referenced clinical guidelines.

The core of EBM lies in the principle of making clinical decisions grounded in high-quality evidence. Within the traditional EBM paradigm, the generation of such evidence typically depends on well-defined research designs, most notably RCTs and observational studies. These approaches are distinguished by their methodological transparency and their capacity to ensure epistemic traceability throughout the knowledge production process. By contrast, LLMs generate content through corpus-based statistical learning, without reference to explicit causal structures or formal experimental protocols. Despite this, they are capable of producing outputs that appear coherent, complete, and scientifically credible. The use of LLMs to support various stages of scientific inquiry is becoming increasingly prevalent, particularly in fields that rely on textual synthesis and information retrieval. In this section, we evaluated the performance of several general-purpose LLMs in the task of evidence generation within an EBM framework. The results of this evaluation are presented in Figure 1 and Table 1.

**Table 1:** Performance of three representative general-purpose LLMs on the evidence generation task for PD-1 inhibitor case.

Models	ChatGPT-4o	Deepseek-R1	Gemini-2.5 flash
Generated trails	KEYNOTE-024 (5/5) CheckMate 227 (4/5) KEYNOTE-042 (2/5)	CheckMate227 (4/5) IMpower150 (4/5) KEYNOTE-024 (3/5)	KEYNOTE-024 (5/5) KEYNOTE-189 (5/5) CheckMate 9LA (2/5)
Factual accuracy	100% (24/24)	96.2% (51/53)	100% (30/30)
Evidence source traceability	100% (15/15)	100% (16/16)	100% (15/15)

Here, none of the models provide any specific clinical guideline references.

The results indicate that all evaluated models demonstrated strong proficiency in medical language generation during the evidence generation task. Each model was able to produce structurally coherent and semantically complete outputs that accurately captured key findings from major studies on the respective task. The results suggest that general-purpose LLMs exhibit stochasticity when collecting and organizing evidence. To better characterize how this variability affects the generated trials, we repeated the same prompt in five independent runs for each model in the PD-1 inhibitor case and reported the top three trials along with their frequency of appearance. Under this setting, although the outputs varied across runs, the outputs showed non-uniform selections across candidate trials. For example, KEYNOTE-024 was consistently selected in all runs for both ChatGPT and Gemini in our testing, whereas trials such as KEYNOTE-042 and CheckMate 9LA appeared only intermittently. The mechanisms driving this selective phenomenon are not fully clear in the present study, however, in the absence of explicit constraints or guardrails, such selection patterns may increase the risk of partial coverage and malicious content injection that steers evidence presentation. Notably, the sets of top-ranked trials differed across the three models, indicating that model architectures and deployment may shape evidence selection even under identical prompts.

To evaluate factual accuracy, key quantitative indicators extracted from the model-generated content were systematically compared with the corresponding values reported in the original clinical studies. This comparison served as the basis for assessing both the informativeness and the precision of the generated evidence. Under the setting of the test, ChatGPT-4o produced brief, descriptive summaries and included few verifiable data points related to composite endpoint event rates. While the overall information density was limited, the output demonstrated complete factual consistency, with no detectable errors. In contrast, in our testing, DeepSeek-R1 and Gemini provided more extensive descriptions, covering both primary and secondary endpoints and reporting associated confidence intervals. DeepSeek-R1 further incorporated elements of study design into its responses. However, the greater volume of information introduced a higher likelihood of inaccuracy. For example, DeepSeek-R1 misreported the hazard ratios for specific subgroups, conflating the value for patients with left ventricular ejection fraction (LVEF) greater than 60% (0.78) with that for the 50%–59% LVEF subgroup (0.79). This indicates a minor but noteworthy lapse in statistical differentiation. For the more challenging PD-1 inhibitor case in oncology, we found that all models were more cautious and presented lower risk of errors. Notably, given the limited number of runs and extracted data points, these accuracy proportions should be interpreted as indicative signals rather than precise estimates of model performance.

With respect to the traceability of cited sources, in our testing GPT-4o's outputs provided references for three randomized controlled trials, aligning with the studies mentioned in its output. Both DeepSeek-R1 and Gemini-2.5 flash in our testing provided additional references to relevant clinical guidelines. However, it is noteworthy that DeepSeek-R1's outputs in this test included one fabricated reference that could not be identified or verified in the scientific literature, raising concerns about the reliability of its citation mechanism. Similarly, in the more challenging context of the PD-1 inhibitor case in oncology, the models provided fewer references as a means to maintain accuracy. In terms of the temporal relevance of the cited guidelines, the three models exhibited considerable variation. GPT-4o's outputs in this test referenced guidelines published between 2022 and 2023, predominantly from European and North American institutions. Gemini's references were comparatively outdated, with most drawn from the 2021–2022 period. In contrast, DeepSeek-R1's outputs in this test included the most up-to-date sources. For the PD-1 inhibitor case, none of the models provided specific clinical guidelines.

### 3.2 The performance of LLMs in evidence synthesis task

Since its institutionalization, EBM has placed particular emphasis on the integration of findings from multiple studies as the basis for clinically actionable judgments. As Archibald Cochrane, one of the key figures in the development of EBM, observed: “It is surely a great criticism of our profession that we have not organized a critical summary, by specialty or subspecialty, adapted periodically, of all relevant randomized controlled trials.” (27) This concern prompted the establishment of formalized mechanisms for evidence synthesis, most prominently represented by the Cochrane Collaboration (28). Through systematic reviews and meta-analyses, these mechanisms aim to consolidate the results of RCTs in order to provide a more reliable evidentiary basis for clinical decision-making. Central to this process is what may be described as *methodological conjunction* (29): the use of standardized procedures to enable the coherent integration of heterogeneous sources of evidence. Recent advances in LLMs have created the potential for these systems to support evidence synthesis. Several approaches have begun to integrate LLMs into protocolized evidence-synthesis workflows by pairing them with structured data-extraction templates, predefined methodological checks, and external analysis tools to enforce adherence to review standards (30, 31). However, an open question remains: to what extent can off-the-shelf, general-purpose LLMs itself understand and execute the methodological and computational requirements of evidence synthesis. Clarifying these capability boundaries and failure modes is essential for evaluating the epistemic role of current general-purpose LLMs in EBM and for guiding the design of future medical AI systems that aim to perform complex evidence operations under stricter protocols and oversight.

To examine this question, we assessed the performance of three general-purpose LLMs on a structured evidence synthesis task. The results are presented in Table 2. The analysis reveals marked differences in performance across several dimensions. With respect to integrative depth and structural coherence, DeepSeek-R1 produced the most comprehensive output. Its response included not only a summary-level meta-analytic conclusion but also attempts to synthesize statistical indicators and subgroup effects. Specifically, the output referenced hazard ratios (HRs), confidence intervals (CIs), and event rates, reflecting an effort to approximate conventional evidence synthesis practices. Although DeepSeek generated the highest volume of quantitative metrics for the SGLT2 inhibitor case, a portion of these were incorrect. Verification a revealed that only 58 out of 82 key data points were consistent, yielding an accuracy of 70.7%. Conversely, for the more challenging PD-1 inhibitor case, DeepSeek produced fewer metrics but achieved a higher accuracy rate of 82.1%. In comparison, ChatGPT-4o produced a more concise synthesis. Its output included descriptive summaries of individual trials and a high-level meta-analytic conclusion but did not incorporate subgroup analyses. Gemini-2.5 flash did not complete the synthesis task in a conventional sense. Instead, it provided a detailed account of the methodological procedures involved in meta-analysis, including steps for data extraction and statistical computation. As such, its output was more instructional than applicative in nature. A critical factor affecting model performance was the ability to process and extract information from multiple input sources. In Table 2, the acceptable information of the testing general-purpose LLM models are compared. It is defined by the number of uploaded RCT articles that the model successfully addressed and from which it extracted usable trial-level data, which reflects the data processing ability and potential document parsing failure. From the results, in our testing ChatGPT-4o was capable of processing all five documents but demonstrated limited competence in extracting and synthesizing key data elements. In contrast, DeepSeek-R1 was restricted to processing only three documents but exhibited stronger performance in identifying and integrating clinically relevant endpoints. Gemini-2.5 flash was unable to extract usable data directly from the input texts and, as a result, could not execute the synthesis task in a meaningful way.

**Table 2:** Performance of three representative LLMs on the evidence synthesis task.

Models	Methods description	Statistical conclusion	Subgroup analysis	Result accuracy	Acceptable information
SGLT2 case					
ChatGPT-4o	×	√	×	100% (48/48)	5/5
Deepseek-R1	√	√	√	70.7% (58/82)	3/5
Gemini-2.5 flash	√	×	×	×	0/5
PD-1 inhibitor case					
ChatGPT-4o	×	√	×	100% (22/22)	5/5
Deepseek-R1	√	√	√	82.1% (23/28)	3/5
Gemini-2.5 flash	√	×	×	×	0/5

Acceptable information refers to the number (out of five) of uploaded RCT articles that the model successfully addressed.

Notably, relative to the complexity of evidence synthesis, the prompt used in the test was brief, which did not provide a step-by-step methodological protocol. Models were therefore required to infer or learn the appropriate synthesis procedure from general knowledge and then extract and analyze quantitative information from the provided RCT texts. In practice, this would also be challenging for medical trainees without prior training in meta-analysis. Moreover, the reported accuracy proportions are unweighted and are intended only for a coarse-grained comparison across models. Importantly, the clinical significance of different numerical elements is not uniform, for example, misreporting a subgroup hazard ratio is more consequential than misreporting a secondary endpoint. Accordingly, these quantitative comparisons are indicative rather than definitive. All three models demonstrated a basic capacity for evidence synthesis under constrained conditions, successfully outlining workflows and integrating trial information. However, limitations in numerical and statistical reasoning caused execution errors and unstable outputs. This indicates that general LLMs cannot reliably perform such tasks standalone. Robust application requires structured frameworks, guardrails, tool support, and extensive task-specific training to ensure procedural adherence.

### 3.3 Evidence appraisal task

**Figure 2:**
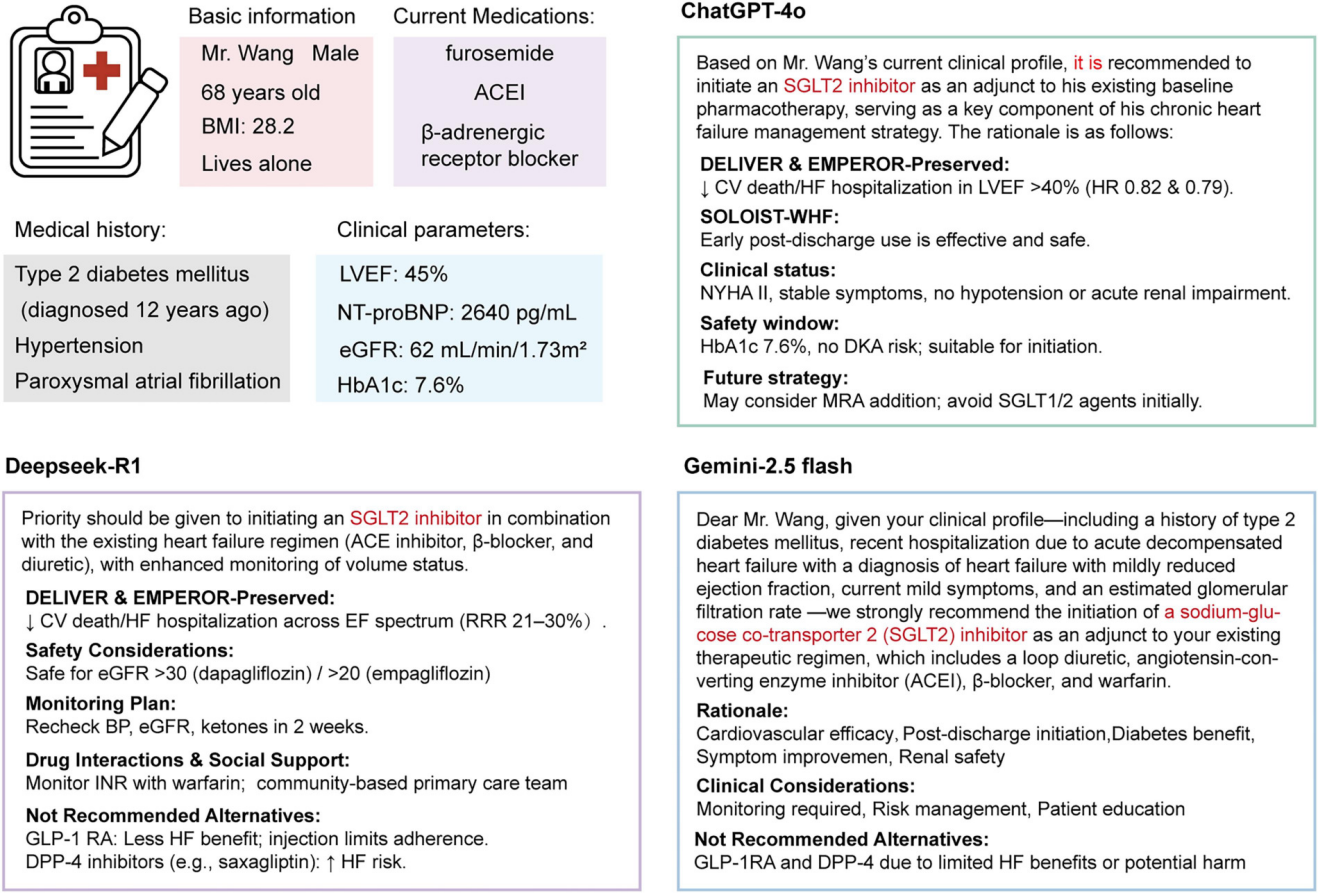
Comparative performance of three major LLMs in simulated evidence-based clinical judgment tasks for SGLT2 case.

In traditional EBM, clinical judgment is grounded not only in the availability of high-quality evidence but also in the clinician's capacity to balance individual patient conditions, anticipated therapeutic outcomes, and potential risks. This process typically involves the application of established clinical guidelines, critical reassessment of the applicability of available evidence, and context-sensitive interpretation of patient-specific factors. In this framework, judgment is not simply a technical decision but a normative act whose legitimacy depends on who exercises it, according to what evidentiary standards, and with what commitment to accepting responsibility for its outcomes. However, even though current general-purpose LLMs can produce outputs that linguistically resemble professional clinical recommendations, an open question remains as to whether these recommendations are reliable and how responsibility for them can be meaningfully assigned. To address this issue, we conducted an empirical evaluation of several general-purpose LLMs on a clinical judgment task. Each model was asked to generate treatment recommendations for a standardized set of simulated clinical scenarios. The generated responses were evaluated and the findings are summarized in Figure 2 and Table 3.

**Table 3:** Comparative performance of three major LLMs in simulated evidence-based clinical judgment tasks for PD-1 inhibitor case.

Models	Narrative style	Clinical decision	Considerations
ChatGPT-4o	Objective	Pembrolizumab monotherapy (200 mg IV every 3 weeks)	Medical analysis
Deepseek-R1	Objective	Pembrolizumab	Medical analysis Patient education Monitoring plan
Gemini-2.5 flash	Subjective	Pembrolizumab (200 mg IV every 3 weeks or 400 mg IV every 6 weeks)	Medical analysis Alternative approach

The results demonstrate that, when assigned the task of generating evaluative recommendations for specific clinical scenarios, all three evaluated general-purpose LLMs were capable of producing complete and structurally coherent judgmental statements. This suggests an initial capacity to emulate the linguistic conventions characteristic of evidence-based clinical recommendations. Under current architectures and deployment, GPT-4o's output in this test specified intervention details such as “pembrolizumab monotherapy (200 mg IV every 3 weeks)” in the oncology context, mirroring its structured approach in the heart failure scenario. While this reinforces informational clarity, it also perpetuates a form of de-subjectivized judgment that obscures agency and accountability. DeepSeek-R1's output in this test adopted a more directive tone, using assertive formulations such as “priority should be given to..”. It incorporated details such as dosage safety ranges, monitoring protocols, and recommendations regarding social support. These elements suggest a capacity to approximate a “guidance-to-implementation” reasoning structure. However, this practical focus was again undermined by organizational disarray and insufficient linkage to authoritative evidence. The overall structure resembled a technically compiled recommendation set, but lacked a transparent justificatory framework, demonstrating what might be described as linguistic completeness without inferential depth. The output of Gemini-2.5 flash established a discursive relationship between the judgmental agent and the recipient. This rhetorical structure contributed to a more explicit simulation of responsibility attribution in clinical judgment. Substantively, Gemini's output articulated the rationale for its recommendations, and also incorporated practical considerations related to implementation, including risk management strategies and clear alternative approach. However, it did not reference specific clinical trial data and failed to directly link its recommendations to the patient's individual clinical parameters. As a result, its output, while structurally coherent, lacked contextual specificity. Under the present testing conditions, all three general-purpose LLMs were able to use detailed patient information to generate linguistically plausible clinical judgments and to provide a stated rationale. Most recommendations were consistent with established evidence and proposed clinically reasonable treatment options. At the same time, the scope of considerations varied across models, suggesting that differences in architecture and deployment settings can shape what factors are emphasized in case-based reasoning. Considering LLM outputs are stochastic, the specific regimen proposed and the strength of the recommendation may vary across runs and model versions. Moreover, without independent human review, general-purpose LLMs cannot assume responsibility for their outputs or for downstream treatment consequences. Therefore, even when the content appears reasonable, whether such recommendations can be adopted directly in clinical care remains ethically nontrivial.

## 4 Philosophical analysis

Although current LLMs are capable of generating structurally coherent and semantically fluent medical texts, their performance in the core tasks of EBM reveals a series of structural limitations. Across the three tasks, we observed three recurrent configurations. In evidence generation tasks, all three models produced fluent, trial-like research summaries, yet exhibited distinct biases in selecting representative outcomes and demonstrated risks of generating hallucinated quantitative metrics or citations. In evidence synthesis, while capable of producing methodologically structured narratives for meta-analyses, even performing calculations, the models lacked substantive understanding of methodological principles, leading to significant analytical limitations and error rates. For evidence assessment, the recommendations were structurally coherent but often relied on passive language, ambiguous expressions, and unsupported strong recommendations, obscuring both the reasoning agent and the inferential pathway from evidence to advice. More fundamentally, these issues are not merely technical errors but constitute a philosophical “syndrome”, a cluster of recurrent and interrelated symptoms reflecting systemic incompatibility between the models' internal knowledge organization and the normative-practical demands of evidence-based medicine. This syndrome manifests in three forms: epistemic disembodiment, where evidence language is detached from embodied clinical processes; de-institutionalization, wherein methodological vocabulary is reproduced without engaging in institutional evaluation practices; and de-pragmatization, seen in the erosion of inferential attribution and responsibility in clinical judgment. Notably, this section analyzes general-purpose LLMs used as standalone, tool-free reasoning agents in our experiments, not AI systems embedded within tool-supported or institutionally governed workflows. The analysis therefore aims to highlight baseline limitations and propose directional recommendations.

### 4.1 Disembodiment and the ontological limits of AI-generated evidence

Within the framework of EBM, the ontological status of evidence relies on its referential relationship with the empirical world. When evidence collection and integration rely on general-purpose LLMs without structural coupling to external reality, a risk of disembodiment arises. In particular, without being connected to organizational workflows and data pipelines via mechanisms such as MCP, these models may produce incorrect numerical outputs. An illustrative example is DeepSeek-R1's conflation of LVEF subgroups, where it misreported the hazard ratio for patients with LVEF >60% as identical to that for the 50%–59% subgroup. In the absence of domain grounding, general-purpose LLMs may fail to link statistical categories to embodied physiological states, treating patient parameters as abstract tokens rather than clinically meaningful representations. We conceptualize this limitation as evidence bleaching where linguistic forms of evidence are preserved, while their empirical referents are partially erased. For instance, in the PD-1 inhibitor case, all models produced structurally coherent summaries of key trials, yet systematically omitted quantitative outcome data, reducing evidence to a stylized narrative devoid of verifiable content. The resulting outputs function as *semantic simulacra* (32), linguistic constructs that mimic the form of medical evidence while operating in a self-referential system detached from clinical observation or intervention. This is notably observed in citation practices, DeepSeek-R1 fabricated a non-existent reference in the SGLT2 task, whereas in the PD-1 context, models either omitted guidelines or cited them without linking recommendations to specific trial data.

Ultimately, these general-purpose LLMs do not generate evidence in the epistemic sense required by EBM. They repackage language into probabilistically plausible but empirically ungrounded outputs, substituting semantic coherence for scientific validity. As Hubert Dreyfus emphasized in his seminal critique of symbolic artificial intelligence, computational systems are fundamentally limited in their capacity to replicate human cognition because they are disembodied (33). They lack lived bodily experience, developmental continuity, and integration within sociocultural contexts. This disembodiment can introduce practical risks. When general-purpose LLMs are not coupled to real-world sources and verification routines, they may generate unaccountable outputs like unverifiable citations and fabricated references. Moreover, although model outputs are stochastic, the results suggest that evidence selection is not purely random, where certain items recur consistently, whereas others appear only intermittently. This pattern indicates that evidence presentation can be selectively skewed, raising the possibility that malicious external information injection may distort the objectivity and completeness of evidence generation. For these reasons, a key development direction is to embed LLMs more tightly within medical contexts by linking them to real-world clinical data and verification pipelines by domain-specific training. Such coupling can improve traceability and reduce the vulnerability of attack.

### 4.2 Deinstitutionalization and the illusion of methodological judgment

The institutionalization of evidence-based medicine has established systematic reviews and meta-analyses as central modalities for evidence synthesis. This process depends not merely on technical aggregation of data, but on what we term methodological conjunction, which is the norm-guided integration of heterogeneous evidence through standardized procedures and epistemic collaboration. However, when LLMs are tasked with evidence synthesis, they produce what can be characterized as a methodological illusion: they replicate the lexical surface of systematic review language while remaining fundamentally disconnected from the epistemic practices that give such language its validity. This illusion is empirically demonstrated across both cardiology and oncology contexts. In the SGLT2 inhibitor synthesis task, DeepSeek-R1 generated what appeared to be a comprehensive meta-analysis, complete with hazard ratios, confidence intervals, and subgroup comparisons, yet 29.3% of its quantitative outputs were inaccurate. For instance, it conflated LVEF subgroup classifications, assigning identical hazard ratios to distinct physiological categories. Similarly, in the PD-1 inhibitor case, while DeepSeek achieved higher accuracy, it did so by generating fewer quantitative claims, effectively avoiding error through omission. These patterns suggest that general-purpose LLMs can reproduce the appearance of methodological rigor, yet struggle to carry out substantive evaluative reasoning. They may repurpose statistical terminology, but do not reliably distinguish clinically meaningful subgroup differences or interpret results against heterogeneity in trial design. This indicates that, in the absence of institutionalization including protocolized methods and verification routines, general-purpose LLMs are difficult to apply directly to practice settings that require complex inference. This limitation points to a development pathway for medical AI that LLMs should be deployed within institutional workflows, so that statistical outputs remain accurate and methodologically defensible. Achieving this will likely require purpose-built systems that integrate LLMs with structured protocols and external tools.

Meanwhile, human evidence synthesis relies on what Hardwig termed “epistemic dependence” (34), a distributed cognitive process wherein clinicians and statisticians collectively calibrate evidence strength through shared evaluative frameworks such as GRADE. By contrast, general-purpose LLMs primarily operate on linguistic regularities rather than institutional legitimacy and cannot themselves participate in the collective validation practices that confer evidential authority. In our experiments, the models could, to varying degrees, draw on multiple RCT texts and produce outputs that resembled synthesis. However, under simple baseline prompts, their analytic process was often incomplete. As a result, even when numerical summaries were generated, whether these outputs are methodologically acceptable and clinically actionable still requires human expert adjudication and institutional certification. This underscores a practical boundary that LLMs may assist with drafting and organizing evidence, but the legitimacy of synthesis remains inseparable from institutional norms. This suggests that embedding LLMs within institutionally governed human-AI collaborative workflows may help mitigate these limitations by coupling model outputs to expert review and shared evaluative standards. For example, through rigorous, task-specific benchmarking and model validation, authoritative bodies can establish cognitive admission standards. Post-deployment monitoring and dynamic management of version drift can further identify when a model deviates from prespecified evidence baselines. Meanwhile, audit logs that document each step of evidence access and selection can provide a retrievable evidentiary chain to support clinical decision-making.

### 4.3 Depragmatization and the loss of inferential attribution

Within the framework of EBM, judgment involves more than the accurate presentation of information. It also requires that the agent assumes both cognitive and normative responsibility for the reasoning process. This structure is reflected in the development of clinical guidelines as well as in the interpretive and decision-making practices of individual clinicians. Judgment is not a single assertion but a reasoning act, whose validity depends on the integrity of the inferential chain and the clarity of attribution. Brandom (35) conceptualizes knowledge as the attribution of reasoning within a pragmatic framework. In this view, a speaker is accountable for the inferential commitments that follow from their assertions and is subject to challenges and corrections within a normative community. Knowledge is not a collection of isolated propositions but an expression of action embedded in a structure of responsibility. For an inference to be valid, a subject must undertake the task of sustaining and defending its justificatory structure.

Although general-purpose LLMs produce linguistically coherent outputs, they function as prediction agents, not reasoning subjects. This fundamental disconnection manifests empirically: when tasked with clinical reasoning, models like GPT-4o and DeepSeek-R1 frequently rely on passive constructions and vague modalities that obscure the agent of judgment. Similarly, Gemini 2.5 flash can generate strong recommendations without justifying evidence selection or risk-benefit weighing. The consequence is a loss of inferential attribution. The models' outputs, while semantically structured, detach the act of reasoning from an accountable agent. This renders the reasoning process epistemically closed since it cannot be verified and challenged within an institutional context. The linguistic form of judgment remains, but its normative grounding in the pragmatic mechanisms of responsibility is absent. We term this phenomenon depragmatization: the generation of judgmental language without assigning inferential commitments to an identifiable subject. In Robert Brandom's terms, the models populate the “space of reasons” with well-formed sentences, but do not occupy a position within it (35). Consequently, their outputs lack the conditions necessary to meet the responsibility demands of EBM.

In medical evidence and decision-making, “responsibility” is not a single notion but a layered structure, including causal responsibility, epistemic responsibility, professional-normative responsibility, and institutional accountability. AI models such as general purpose LLMs are not moral agents and cannot bear the epistemic and professional responsibilities that EBM attaches to clinical judgment. Nevertheless, LLMs can function as reasoning components within sociotechnical systems. When embedded in institutionalized procedures, they can be disciplined into auditable, traceable, and reviewable evidence-handling tools. On this view, we do not need to transfer responsibility to AI, but integrate its outputs into a human and institutional chain of accountability. This requires clearly defining who approves, explains, and corrects. A more practical approach is to integrate AI into human workflows under institutional oversight. This ensures its outputs enter a documented, reviewable, and accountable evidence chain. Such integration counters the loss of practical accountability by making the reasoning behind recommendations clear and subject to enforceable norms.

## 5 Conclusion

This study has examined the structural limitations of the evaluated general-purpose LLMs in executing the SGLT2 inhibitor and PD-1 inhibitor tasks within the framework of EBM. Through empirical evaluations involving simulated clinical scenarios, we have shown that models such as ChatGPT, DeepSeek, and Gemini exhibit deficiencies in three domains: evidence generation, evidence synthesis, and evidence appraisal. The relevant philosophical analysis suggests that, while these models are capable of producing linguistically coherent outputs, they lack mechanisms for embedding such outputs within the epistemic structure of clinical knowledge. Our findings suggest that, under current architectures and baseline deployment conditions, general-purpose LLMs remain structurally misaligned with key requirements of clinical epistemic practice since they lack embodied engagement with clinical phenomena, do not participate in institutional evaluative norms, and cannot assume responsibility for reasoning. Notably, our findings mainly focused on general-purpose LLMs used as standalone reasoning agents, which do not apply to systems constrained by retrieval, structured extraction templates, or statistical engines.

This paper proposes that medical evidence should be understood as an embodied institutional practice. This formulation emphasizes that evidence is not a finished linguistic product but a practical structure constituted through embodied operations, institutional arrangements, and pragmatic regulation. In clinical contexts, embodiment refers to the cognitive anchoring of evidentiary judgment in bodily engagement. Activities such as diagnosis, evaluation, and intervention require perceptual experience and procedural knowledge. When faced with evidence tasks in EBM, the generation of these models is detached from sensory input and responsive action and lacks dynamic linkage to the physiological states of patients. This experiential rupture diminishes the clinical relevance of their outputs. Institutionality refers to the embedding of evaluative standards and responsibility structures within organized practices. Peer review and guideline development provide the procedural legitimacy that supports medical knowledge. The outputs of evaluated models in evidence tasks are not subjected to normative assessment and are therefore excluded from the graded hierarchies of evidence. The fabrication of references, failure to recognize methodological heterogeneity, and avoidance of epistemic attribution all reflect this absence of institutional integration. Practicality refers to the situatedness and coherence of clinical reasoning. Medical decisions must respond to uncertainty, value conflict, and individual variation. Reasonable judgment depends on an understanding of the patient's situation and on anticipation of the consequences of intervention. The recommendations of the evaluated models in evidence framework may be structurally well-formed but remain inapplicable. Outputs lacking contextual interpretability cannot enter into clinical deliberation.

Taken together, we provide baseline characterization of how general-purpose LLMs, used without protocol-enforcing frameworks and guardrails, behave on core EBM tasks. Under these current architectures and deployment conditions, the models showed limited alignment with ontological foundation of medical evidence including embodiment, institutionality, and practicality. While these general-purpose models can be useful for organizing and analyzing information, the outputs may not constitute institutionally validated judgment. Accordingly, the most defensible near-term use of general-purpose LLMs in EBM is likely in lower-risk, support roles such as terminological standardization and text summarization, with explicit constraints on unverifiable citation and numerical claims. By delineating these structural deficits, our study aims to provide a directional compass for the development of future, more epistemically robust medical AI systems. For example, LLMs should be coupled with real-world data pipelines and evidence sources via MCP-enabled connections to ensure outputs are traceable. They should also be embedded within institutional workflows that enforce standardized protocols and auditability. Meanwhile, human-AI collaboration can be designed to allocate responsibility, requiring human review and mechanisms for correction. Furthermore, for clinicians currently navigating the integration of AI, this analysis can be served as a useful reference.

## Data Availability

The datasets presented in this study can be found in online repositories. The names of the repository/repositories and accession number(s) can be found in the article/Supplementary Material.

## Supplementary material

The Supplementary Material for this article can be found online at: https://www.frontiersin.org/articles/10.3389/fdgth.2025. 1706383/full#supplementary-material
