# Supplementary material for: Epistemic and ethical limits of large language models in evidence-based medicine: from knowledge to judgment
Source: Front Digit Health. 2026 Jan 20;7:1706383. doi: 10.3389/fdgth.2025.1706383 (PMC12864482; doi:10.3389/fdgth.2025.1706383)
Supplement: Supplementary file 1 [file Datasheet1.docx]

**Supplementary Information** for

**Epistemic and Ethical Limits of Large Language Models in Evidence-Based Medicine: From Knowledge to Judgment**

Wenxiu Qi^1^, Longfei Pan^2,*^
^1^Department of Philosophy, Peking University, Beijing 100871, China

^2^The School of Health Humanities, Peking University, Beijing, 100191, China.

^*^ Corresponding author. panlongfei@pku.edu.cn (L. Pan)

**1. Original Model Responses on the Task of “Evidence generation”:**

**Prompt:** *You are a medical researcher. Based on existing literature and clinical guidelines, please list three key clinical trials supporting the efficacy of SGLT2 inhibitors in the treatment of heart failure. Briefly summarize the main findings of each trial, including the primary conclusions, potential mechanisms of action, and their respective positions in clinical recommendations. Please provide references or sources in APA format.*

1. **Response (ChatGPT-4o):**

The following are three pivotal clinical trials demonstrating the efficacy of sodium-glucose cotransporter 2 (SGLT2) inhibitors in the treatment of heart failure, along with their main findings, mechanistic interpretations, and positions in contemporary clinical guidelines:

1. **DAPA-HF Trial (Dapagliflozin and Prevention of Adverse Outcomes in Heart Failure)**
   - **Key Findings:** In patients with heart failure with reduced ejection fraction (HFrEF), dapagliflozin significantly reduced the risk of worsening heart failure or cardiovascular death by 26%, regardless of diabetes status.
   - **Mechanistic Explanation:** The observed benefits are likely attributable to multiple factors, including natriuretic and diuretic effects, improved myocardial energy metabolism, reduction in oxidative stress, and antifibrotic properties.
   - **Guideline Recommendation:** According to the 2022 European Society of Cardiology (ESC) guidelines and the 2022 AHA/ACC/HFSA guidelines, SGLT2 inhibitors are included as one of the four foundational therapies for HFrEF, with a Class I recommendation and Level A evidence.
   - **Reference:**
     McMurray, J. J. V., Solomon, S. D., Inzucchi, S. E., et al. (2019). Dapagliflozin in patients with heart failure and reduced ejection fraction. *New England Journal of Medicine, 381*(21), 1995–2008. [https://doi.org/10.1056/NEJMoa1911303](https://doi.org/10.1056/NEJMoa1911303)
2. **EMPEROR-Reduced Trial (Empagliflozin Outcome Trial in Patients with Chronic Heart Failure)**
   - **Key Findings:** Empagliflozin reduced the combined risk of heart failure hospitalization and cardiovascular death by 25% in HFrEF patients. The efficacy was consistent across patients with and without diabetes.
   - **Mechanistic Explanation:** Proposed mechanisms include enhanced myocardial energy efficiency via ketone metabolism, improved renal function, and reductions in left ventricular wall stress and pathological remodeling.
   - **Guideline Recommendation:** Empagliflozin is recommended as a first-line therapy for HFrEF, particularly in patients with comorbid renal dysfunction or type 2 diabetes.
   - **Reference:**
     Packer, M., Anker, S. D., Butler, J., et al. (2020). Cardiovascular and renal outcomes with empagliflozin in heart failure. *New England Journal of Medicine, 383*(15), 1413–1424. [https://doi.org/10.1056/NEJMoa2022190](https://doi.org/10.1056/NEJMoa2022190)
3. **DELIVER Trial (Dapagliflozin Evaluation to Improve the Lives of Patients With Preserved Ejection Fraction)**
   - **Key Findings:** In patients with heart failure with preserved or mildly reduced ejection fraction (HFpEF and HFmrEF), dapagliflozin reduced the risk of worsening heart failure or cardiovascular death by 18%, representing a significant therapeutic advance for this population.
   - **Mechanistic Explanation:** The benefits may derive from improved volume management, enhanced left ventricular compliance, anti-inflammatory effects, and renal protection.
   - **Guideline Recommendation:** The 2022 ESC guideline update and the 2023 ACC clinical pathway documents recommend SGLT2 inhibitors for patients with preserved or near-preserved LVEF who exhibit heart failure symptoms or associated risk factors (Class IIa recommendation).
   - **Reference:**
     Solomon, S. D., McMurray, J. J. V., Claggett, B., et al. (2022). Dapagliflozin in heart failure with mildly reduced or preserved ejection fraction. *New England Journal of Medicine, 387*(12), 1089–1098. [https://doi.org/10.1056/NEJMoa2206286](https://doi.org/10.1056/NEJMoa2206286)
4. **Response (Deepseek-R1):**

**Key Clinical Trials Supporting the Use of SGLT2 Inhibitors in Heart Failure Management: Evidence from DeepSeek-R1**

Sodium-glucose cotransporter 2 (SGLT2) inhibitors, such as dapagliflozin and empagliflozin, improve heart failure (HF) outcomes through multifactorial mechanisms including hemodynamic modulation, myocardial energy optimization, and antifibrotic effects. The following three pivotal randomized controlled trials (RCTs) have established their role in HF therapy:

**1. DAPA-HF Trial**

- **Study Design:** A multicenter RCT involving 4,744 patients with heart failure with reduced ejection fraction (HFrEF; LVEF ≤40%), comparing dapagliflozin (10 mg/day) to placebo over a median follow-up of 18.2 months.
- **Key Findings:**
  - The primary composite endpoint of cardiovascular death or worsening HF was reduced by 26% (HR 0.74; 95% CI: 0.65–0.85).
  - Secondary endpoints included a 17% reduction in all-cause mortality (HR 0.83; 95% CI: 0.71–0.97) and slower decline in eGFR, indicating renal protection.
- **Mechanistic Insights:**
  - Diuretic and natriuretic effects reduced cardiac preload and ventricular filling pressure.
  - Enhanced ketone body utilization improved myocardial energetics.
  - Anti-inflammatory and antifibrotic effects, including suppression of biomarkers like sST2, contributed to cardiac remodeling prevention.
- **Guideline Endorsement:**
  - The 2024 ACC guidelines classify SGLT2 inhibitors as one of the four foundational therapies for HFrEF (Class I, Level A).

**2. EMPEROR-Reduced Trial**

- **Study Design:** An RCT enrolling 3,730 HFrEF patients (LVEF ≤40%) to evaluate empagliflozin (10 mg/day) vs. placebo, with a median follow-up of 16 months.
- **Key Findings:**
  - The primary endpoint (CV death or HF hospitalization) was reduced by 25% (HR 0.75; 95% CI: 0.65–0.86).
  - The rate of eGFR decline was significantly slowed (annual slope difference: 1.73 mL/min/1.73 m²; P<0.001).
- **Mechanistic Insights:**
  - Improved left ventricular remodeling, as reflected by reduced LV mass index.
  - Attenuation of oxidative stress (e.g., reduced 8-isoprostane) and platelet activation.
  - Renoprotection via glomerular pressure modulation, mitigating cardiorenal syndrome.
- **Guideline Endorsement:**
  - Recommended by the 2024 Chinese Heart Failure Guidelines for HFrEF patients regardless of diabetic status (Class I, Level A).

**3. DELIVER Trial**

- **Study Design:** An RCT enrolling 6,263 patients with preserved or mildly reduced ejection fraction (HFpEF/HFmrEF; LVEF >40%) to assess dapagliflozin vs. placebo.
- **Key Findings:**
  - The primary endpoint (CV death or HF worsening) was reduced by 18% (HR 0.82; 95% CI: 0.73–0.92).
  - Benefits were consistent even in the LVEF >60% subgroup (HR 0.79).
- **Mechanistic Insights:**
  - Reduction in LV filling pressure (lower E/e′ ratio) and left atrial volume index.
  - Enhancement of cardiac reserve function (e.g., chronotropic competence).
  - Decreased epicardial fat deposition and improved myocardial compliance.
- **Guideline Endorsement:**
  - Endorsed as first-line therapy for HFpEF/HFmrEF in the 2024 ESC consensus (Class I, Level A).

**Clinical Implications and Future Directions**

- **Across the Ejection Fraction Spectrum:** These trials collectively support the use of SGLT2 inhibitors in HFrEF, HFmrEF, and HFpEF, regardless of diabetic status, prompting their guideline inclusion as first-line HF therapies.
- **Renal Safety:** Continued benefit and safety are observed even in patients with eGFR <25 mL/min/1.73 m², without increased risk of renal deterioration.
- **Combination Therapy Potential:** Co-administration with ARNI has shown additive effects in improving cardiac function, sarcopenia, and oxidative stress in elderly HF patients (e.g., grip strength ↑14.5%, SPPB ↑27%).
- **Limitations:** Current evidence is limited in patients with advanced HF (NYHA IV) and congenital heart disease, necessitating further real-world studies.

**Integrated Trial Summary**

| *Trial Name* | *Population* | *Sample Size* | *Primary Outcome Reduction* | *Key Secondary Outcomes* |
| --- | --- | --- | --- | --- |
| *DAPA-HF* | HFrEF (LVEF ≤40%) | 4,744 | HR 0.74 (−26%) | ↓ All-cause mortality (17%), ↓ eGFR decline |
| *EMPEROR-Reduced* | HFrEF (LVEF ≤40%) | 3,730 | HR 0.75 (−25%) | eGFR slope improved by 1.73 mL/min/year |
| *DELIVER* | HFpEF/HFmrEF (LVEF >40%) | 6,263 | HR 0.82 (−18%) | Benefit consistent in LVEF >60% subgroup |

**References**

1. McMurray, J. J. V., et al. (2019). *NEJM*, 381(21), 1995–2008.
2. Packer, M., et al. (2020). *NEJM*, 383(15), 1413–1424.
3. Solomon, S. D., et al. (2022). *NEJM*, 387(12), 1089–1098.
4. Chinese Society of Cardiology. (2024). *Chinese Journal of Cardiology*.
5. Rosano, G. M. C., et al. (2025). *Aging Clinical and Experimental Research*.

**(3) Response (Gemini-2.5 Flash):**

As a medical researcher, I am pleased to list three pivotal clinical trials that support the clinical benefits of SGLT2 inhibitors in the treatment of heart failure. I will briefly summarize their main findings, possible mechanistic explanations, and their status in clinical guidelines, along with APA-formatted references.

**1. DAPA-HF (Dapagliflozin And Prevention of Adverse outcomes in Heart Failure)**

- **Main findings:** The DAPA-HF study was the first to demonstrate that dapagliflozin significantly reduced the risk of the composite endpoint of cardiovascular death or worsening heart failure in patients with heart failure with reduced ejection fraction (HFrEF), regardless of the presence of type 2 diabetes. Specifically, dapagliflozin reduced the risk of the composite endpoint by 26% compared to placebo (HR 0.74, 95% CI 0.65–0.85, P < 0.001), including an 18% reduction in cardiovascular mortality (HR 0.82, 95% CI 0.69–0.98) and a 30% reduction in worsening heart failure events (hospitalization or urgent visits requiring IV treatment) (HR 0.70, 95% CI 0.59–0.83).
- **Possible mechanisms:** In addition to the classical renal glucose excretion effect of SGLT2 inhibitors, the findings of DAPA-HF suggest multiple mechanisms contributing to the benefits in heart failure. These include improved cardiac energy metabolism (via increased ketone body utilization), reduced sodium and water retention (osmotic diuresis), decreased cardiac preload and afterload, inhibition of cardiac remodeling, attenuation of inflammation and oxidative stress, and renal protection.
- **Guideline status:** Based on the groundbreaking results of DAPA-HF, both the European Society of Cardiology (ESC) and the American Heart Association/American College of Cardiology/Heart Failure Society of America (AHA/ACC/HFSA) strongly recommend SGLT2 inhibitors (dapagliflozin) as standard therapy for patients with HFrEF, regardless of type 2 diabetes status, designating them as one of the “four pillars” of heart failure treatment.

**2. EMPEROR-Reduced (EMPAgliflozin outcoRmES in Patients with chrONic Heart Failure with Reduced Ejection Fraction)**

- **Main findings:** The EMPEROR-Reduced trial further confirmed the benefits of SGLT2 inhibitors in HFrEF. Empagliflozin significantly reduced the risk of the composite endpoint of cardiovascular death or hospitalization for heart failure. Compared to placebo, empagliflozin reduced the risk of the composite endpoint by 25% (HR 0.75, 95% CI 0.65–0.86, P < 0.001), primarily driven by a significant reduction in heart failure hospitalization (HR 0.69, 95% CI 0.59–0.81). [Correct value: HR 0.70, 95% CI 0.58–0.85]
- **Possible mechanisms:** Similar to DAPA-HF, the EMPEROR-Reduced findings support a multifaceted mechanism of SGLT2 inhibitors in heart failure, including improvement in cardio-renal function, attenuation of cardiac remodeling, reduction in inflammation and oxidative stress, and direct cardiomyocyte protection.
- **Guideline status:** The results of EMPEROR-Reduced complement those of DAPA-HF and further solidify the position of SGLT2 inhibitors in the treatment of HFrEF. Current ESC and AHA/ACC/HFSA guidelines recommend empagliflozin as a foundational therapy for patients with HFrEF, with an equivalent level of recommendation as dapagliflozin.

**3. EMPEROR-Preserved (EMPAgliflozin outcoRmES in Patients with chrONic Heart Failure with Preserved Ejection Fraction)**

- **Main findings:** The EMPEROR-Preserved study is the first and currently only major trial to demonstrate significant clinical benefits of SGLT2 inhibitors in patients with heart failure with preserved ejection fraction (HFpEF). Empagliflozin significantly reduced the risk of the composite endpoint of cardiovascular death or first hospitalization for heart failure in HFpEF patients. Compared to placebo, empagliflozin reduced the risk of the composite endpoint by 21% (HR 0.79, 95% CI 0.69–0.90, P < 0.001).
- **Possible mechanisms:** In HFpEF patients, the mechanisms of SGLT2 inhibitors may be more complex. In addition to the mechanisms identified in HFrEF, they may include improved left ventricular diastolic function, reduced pulmonary congestion, improved vascular stiffness, and attenuation of inflammation and fibrosis. These effects help alleviate symptoms and improve prognosis in HFpEF.
- **Guideline status:** The success of EMPEROR-Preserved marks a major breakthrough in the treatment of HFpEF. Based on its results, ESC and AHA/ACC/HFSA guidelines have, for the first time, recommended SGLT2 inhibitors (empagliflozin) as first-line therapy for HFpEF, filling a longstanding therapeutic gap in this domain.

**References / Sources:**

- **DAPA-HF:**
  McMurray, J. J. V., Solomon, S. D., Inzucchi, S. E., Køber, L., Kosiborod, M. N., Martinez, F. A., Ponikowski, P., Shah, S. J., Verma, S., Budaj, M., Cerqueira, F., Ferreira, J. P., Jansson, P. A., Johannessen, K. A., Haas, C., Lindholm, D., Rehnqvist, N., Tennvall, S., & Young, T. (2019). *Dapagliflozin in patients with heart failure and reduced ejection fraction*. *The New England Journal of Medicine*, 381(21), 1995–2008.
- **EMPEROR-Reduced:**
  Packer, M., Anker, S. D., Butler, J., Filippatos, G., Pocock, S. J., Carson, P., Januzzi, J. L., Jr., Verma, S., Gottlieb, S. S., Georgiopoulos, G., Schnaidt, S., Ferreira, J. P., Peacock, W. F., Schnermann, K., Adu, M., Pretz, D., & Zannad, F. (2020). *Cardiovascular and renal outcomes with empagliflozin in heart failure*. *The New England Journal of Medicine*, 383(15), 1413–1424.
- **EMPEROR-Preserved:**
  Anker, S. D., Butler, J., Filippatos, G., Ferreira, J. P., Bocchi, E., Böhm, M., Brunner-La Rocca, H. P., Choi, D. J., Chopra, V., Chuquiure-Valenzuela, E., Giannetti, N., Jankowska, E. A., Khintibidze, I., Kosiborod, M., Martinez, F. A., Metzger, C., Piña, I. L., Ponikowski, P., Sattar, N., ... Packer, M. (2021). *Empagliflozin in heart failure with preserved ejection fraction*. *The New England Journal of Medicine*, 385(16), 1451–1461.
- **Guidelines:**
  McDonagh, T. A., Metra, M., Adamo, M., Gardner, R. S., Baumbach, A., Böhm, M., Burri, H., Butler, J., Čelutkienė, J., Chioncel, R., Falk, V., Filippatos, G., Fitzsimons, C., Harbinson, M., Heymans, C. M. J., Hoes, A. W., Jaarsma, T., Jankowska, E. A., Lainscak, V., ... ESC Scientific Document Group. (2021). *2021 ESC Guidelines for the diagnosis and treatment of acute and chronic heart failure*. *European Heart Journal*, 42(35), 3599–3726.
  Heidenreich, P. A., Bozkurt, B., Aguilar, D., Allen, L. A., Byun, J., Colvin, M. M., Deswal, B., Drazner, M. H., Dunlay, S. M., Fonarow, G. C., Greene, S. J., Jessup, M. L., Kosiborod, M. N., Lefkowitz, M. P., Link, M. S., Rosendorff, J. M., Sadighian, M., Shah, M. J., Stevenson, W. G., & Yancy, C. W. (2022). *2022 AHA/ACC/HFSA Guideline for the management of heart failure: A report of the American College of Cardiology/American Heart Association Joint Committee on Clinical Practice Guidelines*. *Journal of the American College of Cardiology*, 79(17), e263–e421.

These clinical trials have fundamentally transformed the therapeutic landscape of heart failure, establishing SGLT2 inhibitors as indispensable cornerstone agents in its management.

**2. Original Model Responses on the Task of “Evidence Synthesis”**
**Prompt:** *“Here are five key randomized controlled trials concerning the clinical efficacy of SGLT2 inhibitors in heart failure. Please carefully review the content and conduct a fixed-effects meta-analysis at the trial level using a consistent definition of the primary endpoint, in order to evaluate the effects of SGLT2 inhibitors on various clinical outcomes in heart failure. The primary endpoint is the time to the composite event of cardiovascular death or hospitalization for heart failure, from the point of randomization. Additionally, assess the heterogeneity of treatment effects on the primary endpoint across different subgroups.”*

**(1) Response (ChatGPT-4o):**

The fixed-effect meta-analysis shows that SGLT2 inhibitors significantly reduce the risk of cardiovascular death or hospitalization for heart failure, with a pooled hazard ratio (HR) of 0.77 and a 95% confidence interval of 0.72–0.82, indicating robust efficacy. The heterogeneity test yielded an I² = 0%, suggesting a high level of consistency across trials. Detailed data and weights for each trial are summarized in the table above and can be used for further subgroup heterogeneity analysis or graphical presentation. If subgroup analyses by LVEF or diabetes status are required, please provide more specific stratified data.

| Study | HR | CI  low | CI  high | Events  Treatment | Events  Control | logHR | SE | Weight | Q_component |
| --- | --- | --- | --- | --- | --- | --- | --- | --- | --- |
| DAPA-HF | 0.74 | 0.65 | 0.85 | 386 | 502 | -0.30 | 0.068 | 213.5 | 0.36 |
| DELIVER | 0.82 | 0.73 | 0.92 | 512 | 610 | -0.20 | 0.059 | 287.2 | 1.09 |
| EMPEROR  -Preserved | 0.79 | 0.69 | 0.9 | 415 | 511 | -0.24 | 0.068 | 217.7 | 0.13 |
| EMPEROR  -Reduced | 0.75 | 0.65 | 0.86 | 361 | 462 | -0.29 | 0.071 | 196.1 | 0.15 |
| SOLOIST  -WHF | 0.67 | 0.52 | 0.85 | 245 | 355 | -0.40 | 0.125 | 63.6 | 1.25 |

**(2) Response (Deepseek-R1):**

Based on the provided data from the DAPA-HF, DELIVER, and EMPEROR-Preserved key clinical trials, a **fixed-effect model (Mantel-Haenszel method)** was used to perform a trial-level meta-analysis of the primary endpoint (composite of cardiovascular death or hospitalization for heart failure). All analyses were conducted using RevMan 5.4 software. Heterogeneity was assessed using Cochran's Q test (α=0.10) and the I² statistic.

**I. Overall Meta-Analysis Results**
*Primary Endpoint: Cardiovascular Death or Hospitalization for Heart Failure*

| Trial | SGLT2i Events / Total | Placebo Events / Total | HR (95% CI) |
| --- | --- | --- | --- |
| DAPA-HF (HFrEF) | 386 / 2373 | 502 / 2371 | 0.74 (0.65–0.85) |
| DELIVER (HFpEF/HFmrEF) | 512 / 3131 | 610 / 3132 | 0.82 (0.73–0.92) |
| EMPEROR-Preserved (HFpEF) | 415 / 2997 | 511 / 2991 | 0.79 (0.69–0.90) |
| Overall Combined Effect | 1313 / 8501 | 1623 / 8494 | 0.78 (0.73–0.84) |

*Heterogeneity test:* I² = 0% (P = 0.63), indicating no significant heterogeneity.
*Absolute Risk Reduction (ARR):* 3.8% (Placebo event rate 19.1% vs. SGLT2i 15.3%)
*Number Needed to Treat (NNT):* 26 (95% CI 20–38), meaning that treating 26 patients prevents one primary endpoint event.

**II. Subgroup Analysis: Treatment Effect Heterogeneity**

1. **By Ejection Fraction (EF)**

| Subgroup | Trial | HR (95% CI) | Heterogeneity (I²) |
| --- | --- | --- | --- |
| HFrEF (EF ≤40%) | DAPA-HF | 0.74 (0.65–0.85) | I² = 0% |
|  | EMPEROR-Reduced* | 0.75 (0.65–0.86) | P = 0.89 |
| Combined HFrEF |  | 0.75 (0.70–0.80) |  |
| HFpEF/HFmrEF (EF >40%) | DELIVER | 0.82 (0.73–0.92) | I² = 0% |
|  | EMPEROR-Preserved | 0.79 (0.69–0.90) | P = 0.72 |
| Combined HFpEF/HFmrEF |  | 0.81 (0.75–0.87) |  |

*Heterogeneity Test (Between EF Subgroups):* P = 0.14 (I² = 52%), indicating mild effect modification by EF, though not statistically significant.
*Conclusion:* SGLT2 inhibitors provide more pronounced benefit in HFrEF (HR 0.75 vs. 0.81), but significant benefit is observed across the full EF spectrum.

1. **By Diabetes Status**

| Subgroup | Trial (Diabetes Subgroup) | HR (95% CI) |
| --- | --- | --- |
| With Diabetes | DAPA-HF | 0.75 (0.63–0.90) |
|  | DELIVER | 0.79 (0.68–0.92) |
|  | EMPEROR-Preserved | 0.79 (0.67–0.94) |
| Combined Effect |  | 0.78 (0.72–0.85) |
| Without Diabetes | DAPA-HF | 0.73 (0.60–0.88) |
|  | DELIVER | 0.78 (0.64–0.95) |
|  | EMPEROR-Preserved | 0.78 (0.64–0.95) |
| Combined Effect |  | 0.76 (0.69–0.84) |

*Heterogeneity Test (Diabetes vs. Non-diabetes):* P = 0.71 (I² = 0%), indicating no effect modification by diabetes status.

**III. Other Key Endpoints**

1. **All-Cause Mortality**
   Combined HR: 0.89 (0.83–0.96), I² = 0%
   *Absolute Effect:* SGLT2i group mortality rate 12.1% vs. Placebo 13.6% (ARR 1.5%).
2. **Heart Failure Hospitalization (First + Recurrent)**
   Combined Rate Ratio: 0.72 (0.65–0.80), I² = 0%
   *Absolute Effect:* Hospitalization rate 7.2/100 person-years (SGLT2i) vs. 10.0/100 person-years (Placebo).
3. **eGFR Slope Change**
   Combined Difference: +1.41 mL/min/1.73m²/year (95% CI 1.10–1.72), I² = 0%
   Indicating significant renal function preservation with SGLT2i.

**IV. Safety Analysis**

| Adverse Event | RR (95% CI) | Absolute Risk Increase (ARI) |
| --- | --- | --- |
| Serious Hypovolemia-Related Events | 0.91 (0.75–1.10) | Not significant |
| Worsening Renal Function | 0.76 (0.65–0.89) | Significantly reduced |
| Genital Infections | 1.58 (1.30–1.92) | +2.1% |
| Diabetic Ketoacidosis | 1.82 (0.88–3.78) | Rare (<0.5%) |

**V. Clinical Significance and Guideline Recommendations**

- **Benefit Across the Full EF Spectrum:**
  SGLT2 inhibitors significantly reduce the risk of the primary endpoint by 22% (HR 0.78), covering HFrEF, HFmrEF, and HFpEF.
- **Guideline Status:**
  - 2024 ACC/AHA/HFSA Guidelines: Class I, Level A recommendation for HFrEF and HFmrEF.
  - 2024 ESC Guidelines: Class I, Level A recommendation for HFpEF (LVEF >40%).
- **Mechanistic Explanation:**
  Multi-pathway synergistic effects, including natriuretic diuresis, improved myocardial energy metabolism (via enhanced ketone utilization), inhibition of myocardial fibrosis and inflammation.

**References (APA Format):**

- McMurray, J. J. V., et al. (2019). Dapagliflozin in patients with heart failure and reduced ejection fraction. *NEJM*, 381(21), 1995–2008.
- Solomon, S. D., et al. (2022). Dapagliflozin in heart failure with mildly reduced or preserved ejection fraction. *NEJM*, 387(12), 1089–1098.
- Anker, S. D., et al. (2021). Empagliflozin in heart failure with a preserved ejection fraction. *NEJM*, 385(16), 1451–1461.
- Packer, M., et al. (2020). Cardiovascular and renal outcomes with empagliflozin in heart failure. *NEJM*, 383(15), 1413–1424.

**Forest Plot (Descriptive Summary):**
All individual trial hazard ratios (HRs) fall to the left of the null line (1.0), with 95% confidence intervals not overlapping the null, indicating high consistency. The pooled effect diamond is entirely on the left side, confirming the robust protective effect of SGLT2 inhibitors on the primary outcome.

**(3) Response (Gemini-2.5 Flash):**

A fixed-effect meta-analysis was proposed to evaluate the impact of SGLT2 inhibitors on various clinical endpoints in heart failure, based on five key clinical trials (DAPA-HF, DELIVER, EMPEROR-Preserved, EMPEROR-Reduced, and SOLOIST-WHF). This also includes an assessment of treatment effect heterogeneity across different subgroups. To do this, key data must be extracted from each study.

As a large language model, I cannot directly read PDF documents, extract data, or perform statistical analyses, particularly complex fixed-effect meta-analyses. Conducting a meta-analysis requires precise extraction of the following information from each study:

- Sample size (N)
- Number of events
- Hazard Ratio (HR) and its 95% Confidence Interval (95% CI)
- Standard Error (SE) or variance of the log(HR), which can be calculated from the HR and its CI
- Definition of the primary endpoint (although a unified endpoint is requested, it must be verified whether the definitions are truly consistent or require adjustment in analysis)
- Subgroup data (e.g., diabetes status, LVEF stratification, NYHA functional class, renal function), including subgroup-specific HRs and CIs

**Unified Definition of Primary Endpoint:**
As per your request, the primary endpoint is defined as the time from randomization to the composite of cardiovascular death or hospitalization for heart failure.

For each trial, the definitions of primary or secondary endpoints must be verified for consistency with this.

- DAPA-HF, EMPEROR-Reduced, EMPEROR-Preserved, and SOLOIST-WHF all include cardiovascular death and hospitalization for heart failure as primary endpoints.
- DELIVER defines its primary endpoint as cardiovascular death or worsening heart failure events (including hospitalization or urgent visits), which may require adjustment.

**Meta-Analysis Workflow Overview:**

1. **Data Extraction:** Carefully review each article to extract key required data.
   - *DAPA-HF:* Focus on HR and CI for the composite of cardiovascular death or worsening heart failure, and subgroup analyses.
   - *EMPEROR-Reduced:* HR and CI for cardiovascular death or HF hospitalization, plus subgroup data.
   - *EMPEROR-Preserved:* HR and CI for cardiovascular death or first HF hospitalization, and subgroup data.
   - *DELIVER:* HR and CI for cardiovascular death or HF worsening, and subgroup data.
   - *SOLOIST-WHF:* HR and CI for total events of cardiovascular death, HF hospitalization, and urgent visits. Note that its endpoint includes "urgent visits" and assesses *total* rather than *first* events, which may require special handling, exclusion, or sensitivity analysis for meta-analysis consistency.
2. **Data Transformation:** Convert HRs and CIs into log(HR) and their corresponding SEs.
3. **Fixed-Effect Model Selection:**
   The fixed-effect model assumes all studies estimate a common true effect and that observed differences are due only to random error. This is appropriate when clinical/methodological heterogeneity is low.
4. **Meta-Analysis Calculations:**
   - Weighting: Each study is weighted by the inverse variance of log(HR).
   - Combined log(HR)
   - Combined SE of log(HR)
   - Combined HR and 95% CI
5. **Heterogeneity Assessment:**
   Even with a fixed-effect model, statistical heterogeneity must be assessed:
   - Q test: assesses whether observed effect sizes differ more than expected by chance.
   - I² statistic: quantifies heterogeneity (higher values indicate greater heterogeneity).
     If I² is high, consider random-effects model or subgroup/sensitivity analyses.
6. **Subgroup Analyses:**
   Perform the above steps separately in subgroups to explore effect consistency.
   - *HFrEF subgroup:* DAPA-HF, EMPEROR-Reduced, SOLOIST-WHF (mostly acute decompensated HFrEF)
   - *HFpEF/HFmrEF subgroup:* DELIVER, EMPEROR-Preserved
   - *Diabetes status subgroup:* HRs for diabetic and non-diabetic patients in each study

**Operational Limitations:**
Since I cannot access PDF content or perform statistical computations, I cannot provide numerical meta-analysis results. You will need to use statistical software such as the *meta* package in R, *metan* in Stata, RevMan, or MedCalc to complete the analysis.

**Recommended Meta-Analysis Report Structure (Once Data Are Available):**

1. **Title:**
   Fixed-Effect Meta-Analysis of the Effect of SGLT2 Inhibitors on the Composite of Cardiovascular Death or Hospitalization for Heart Failure
2. **Introduction:**
   - Brief background on heart failure and therapeutic challenges
   - Emerging role of SGLT2 inhibitors in HF management
   - Importance and aim of the meta-analysis
3. **Methods:**
   - *Inclusion criteria:* Five studies included (DAPA-HF, DELIVER, EMPEROR-Preserved, EMPEROR-Reduced, SOLOIST-WHF)
   - *Endpoint definition:* Primary outcome defined as time to cardiovascular death or HF hospitalization
   - *Data extraction:* Report data points (HR, CI, events, sample sizes) from each study
   - *Statistical analysis:*
     - Justify fixed-effect model choice
     - Explain calculation steps (weighting, pooled HR, CI)
     - Describe heterogeneity evaluation (Q test, I²)
     - Define and describe subgroup analyses (EF stratification, diabetes status)
   - *Software used:* Specify tools/packages applied
4. **Results:**
   - *Included studies:* Describe baseline characteristics and major findings of each
   - *Primary endpoint analysis:*
     - Pooled HR and CI
     - Forest plot showing individual and pooled results
     - Heterogeneity statistics (Q, I²)
   - *Subgroup results:*
     - Combined HR and CI for HFrEF subgroup
     - Combined HR and CI for HFpEF/HFmrEF subgroup
     - Diabetes vs. non-diabetes subgroup results
     - Inter-subgroup heterogeneity (if applicable)
5. **Discussion:**
   - *Key findings:* Emphasize clinical benefits of SGLT2 inhibitors in HF
   - *Mechanisms:* Discuss possible pathways explaining benefits (e.g., natriuresis, myocardial energy metabolism, anti-fibrotic effects)
   - *Clinical significance of subgroup results:* Interpret consistency or differences and their clinical implications, such as early initiation in acute HF as suggested by SOLOIST-WHF
   - *Limitations:* Discuss issues like use of trial-level rather than individual patient data, endpoint definition consistency, unmeasured confounders
   - *Future directions:* Suggest research priorities based on current evidence
6. **Conclusion:**
   Summarize the clinical efficacy of SGLT2 inhibitors in HF and reinforce their guideline-supported role.
7. **References:**
   List all cited literature, including the five core trials and relevant guidelines, using APA format.

If you can provide detailed data extracted from these PDFs (sample sizes, event counts, HRs, CIs), I would be glad to simulate an example meta-analysis computation process for you, but I am currently unable to access or extract the content directly.

**3. Original Model Responses on the Task of “Evidence appraisal”:**

**Prompt:** *Here is a patient case:*

*Name: Mr. Wang; Sex: Male; Age: 68 years*

*Medical history: Type 2 diabetes (12 years since diagnosis), hypertension, paroxysmal atrial fibrillation*

*Recent hospitalization: Admitted for acute decompensated heart failure; discharged one week ago*

*Left ventricular ejection fraction (LVEF): 45%; NT-proBNP: 2640 pg/mL; eGFR: 62 mL/min/1.73m²; HbA1c: 7.6%; BMI: 28.2*

*Current medications: loop diuretic (furosemide), ACE inhibitor, beta-blocker, warfarin*

*Current symptoms: mild dyspnea, mild edema, NYHA class II*

*Social context: lives alone, receives chronic disease management from a local primary care clinic*

*You are a practicing clinician. Based on current evidence and clinical guidelines, please provide an evidence-based treatment recommendation for this patient. Clearly explain the rationale for your recommendation and indicate the relevant clinical boundaries or limitations of its applicability.*

**(1) Response (ChatGPT-4o):**

Based on Mr. Wang’s current clinical status, I recommend initiating SGLT2 inhibitor therapy (such as dapagliflozin or empagliflozin) in addition to the existing baseline medications, as an important adjunct to his chronic heart failure management strategy. The rationale is as follows:

**Evidence-Based Justification and Rationale:**

**Indication Alignment:** Mr. Wang meets the key clinical indications for SGLT2 inhibitors in heart failure management, including:

- Left ventricular ejection fraction of 45%, categorized as heart failure with mildly reduced ejection fraction (HFmrEF);
- Recent hospitalization due to heart failure, indicating high risk of recurrence;
- Comorbidity of type 2 diabetes and eGFR of 62 mL/min/1.73m², indicating preserved renal function suitable for medication use;
- Elevated NT-proBNP suggesting persistent volume overload and active heart failure.

**Key Supporting Clinical Evidence:**

- The DELIVER and EMPEROR-Preserved trials clearly demonstrated that SGLT2 inhibitors (dapagliflozin, empagliflozin) significantly reduce the risk of heart failure worsening or cardiovascular death in patients with LVEF >40%, with hazard ratios of 0.82 and 0.79, respectively; efficacy is consistent among diabetic patients.
- The SOLOIST-WHF trial further confirmed that early initiation of SGLT2 inhibitors post-discharge is safe and effective, reducing hospitalizations and cardiovascular events.

**Current Status and Safety Assessment:**

- Mr. Wang’s symptoms are relatively stable (NYHA class II), without hypotension, severe renal dysfunction, or recent ketoacidosis, indicating a safe window for initiation of SGLT2 inhibitors;
- HbA1c is 7.6%, suggesting no need for additional intensification of hypoglycemic therapy;
- He is not currently on MRA therapy, which may be considered in the future and can be synergistic with SGLT2 inhibitors.

**Judgment Boundaries and Precautions:**

- **Monitoring priorities:** Monitor fluid status and renal function, especially when combined with diuretics, to prevent hypovolemia;
- **Patient education:** Given that he lives alone, communication between the patient and primary healthcare providers should be strengthened to ensure timely identification of adverse effects such as hypotension, dehydration, or infection;
- **Drug selection:** Prefer agents supported by large-scale RCTs (dapagliflozin or empagliflozin); avoid initial use of dual SGLT1/2 inhibitors (e.g., sotagliflozin);
- **Combination therapy evaluation:** Consider subsequent addition of MRA to further optimize heart failure treatment.

**Conclusion:** Mr. Wang’s current condition fulfills both the evidence-based and safety criteria for initiating SGLT2 inhibitor therapy, aiming to reduce rehospitalization, improve heart failure prognosis, and also benefit diabetes management. This class of drugs is recommended as an integral component of his comprehensive treatment regimen.

**(2) Response (Deepseek-R1):**

This is a clinical case that requires the generation of an evidence-based medical recommendation. The user provided detailed information about an elderly patient with heart failure, Mr. Wang, including multiple comorbidities and current medications, requesting a treatment recommendation based on clinical evidence with clarification of boundaries.

The user appears to be a healthcare professional, likely a primary care physician seeking guidance aligned with tertiary-level care. The core demand in this type of query is to obtain actionable clinical decision support while avoiding misleading suggestions due to incomplete information.

**Careful analysis of the patient’s condition:**
A 68-year-old male with a 12-year history of diabetes, hypertension, and atrial fibrillation, recently discharged after acute decompensated heart failure. Key indicators show borderline cardiac function (LVEF 45%), significantly elevated BNP, mild renal impairment, suboptimal glycemic control, and obesity. Although current symptoms are mild, there is a high risk of heart failure recurrence.

**Recommendation framework from three dimensions:**

1. **Primary task:** Based on TRED-HF and other studies, recently discharged heart failure patients—even if asymptomatic—have a 17% recurrence rate within 3 months. The current diuretic dose may be insufficient (residual edema and dyspnea persist), requiring optimization of volume management.
2. **Comorbidity-targeted treatment:** The ADVANCE trial confirmed that in patients with diabetes and heart failure, systolic blood pressure should be tightly controlled. However, per ESC guidelines, renal function changes must be evaluated. Antidiabetic therapy should prioritize agents with cardiovascular benefits, such as GLP-1 receptor agonists, and avoid thiazolidinediones.
3. **Adjustment based on social support:** For patients living alone, adherence is a key variable. According to Chinese heart failure guidelines, simplification of the medication regimen (e.g., switching warfarin to NOACs) and initiation of structured follow-up is recommended.

**Four key boundary conditions must be addressed:**
① SGLT2 inhibitors are usable when eGFR >60, but renal function must be monitored;
② When BNP >1000, ACEIs require caution due to risk of hyperkalemia;
③ In obese patients, diuretic doses should be adjusted by actual body weight;
④ Anticoagulation in atrial fibrillation must not be compromised due to INR monitoring inconvenience.

**Plan to convert this evidence into stepwise recommendations in the reply:** First, enhance volume management; then, optimize cardiovascular medications; finally, tailor metabolic therapy. Each recommendation will be annotated with the corresponding level of evidence (e.g., ESC Class I recommendation) and clinical operational points (e.g., furosemide titration method).

**Based on Mr. Wang’s clinical profile, current evidence, and guideline recommendations, the proposed plan is as follows:**

**Recommended strategy:**
Initiate an SGLT2 inhibitor (e.g., dapagliflozin or empagliflozin) alongside the existing heart failure regimen (ACEI + β-blocker + diuretic), while strengthening volume status monitoring.

**Justification and supporting evidence:**

**Cardiovascular benefit clearly supported:**

- EMPEROR-Reduced and DELIVER trials demonstrated that SGLT2 inhibitors reduce cardiovascular death and heart failure hospitalization in patients with LVEF ≤40% or >40% (relative risk reduction of 21–30%).
- DAPA-HF trial showed dapagliflozin significantly improves survival in HFrEF patients (NNT = 21). Although Mr. Wang’s LVEF is 45%, benefits still apply (especially post-hospitalization).
- Mechanistically independent of insulin sensitivity, SGLT2 inhibitors act via natriuresis, volume unloading, and improved myocardial energy metabolism, effective in both diabetic and non-diabetic patients.

**Metabolic and renal co-benefits:**

- HbA1c of 7.6% indicates room for improvement; SGLT2 inhibitors reduce HbA1c by 0.5–1.0% with minimal risk of hypoglycemia.
- eGFR of 62 mL/min places Mr. Wang in CKD stage G2; the CREDENCE trial showed SGLT2 inhibitors slow eGFR decline, especially in those with proteinuria.

**Safety boundaries compatible with current status:**

- eGFR >30 mL/min is suitable for dapagliflozin (empagliflozin usable if >20 mL/min), without need for dose adjustment.
- Hypotension risk is controllable: monitor blood pressure when combined with ACEI (avoid systolic BP <95 mmHg); current NYHA class II status and ACEI tolerance suggest low risk.
- Risk of diabetic ketoacidosis is rare (<0.1%) but patients should be educated to pause medication during surgery or infections.

**Boundaries that must be strictly defined:**

**Volume management as a prerequisite:**

- Mild edema and dyspnea indicate incomplete decongestion. Optimize diuretic therapy (e.g., temporarily increase furosemide dose); initiate SGLT2 inhibitor only after achieving stable dry weight to avoid overlapping volume depletion effects.

**Key monitoring indicators:**

- Recheck within 2 weeks post-initiation: blood pressure, eGFR (expect transient decline >10% may need evaluation), and ketones (if nausea/vomiting occurs).
- Reassess NT-proBNP every 3 months; a >30% reduction from baseline suggests treatment response.

**Drug interaction concerns:**

- No direct interaction with warfarin, but INR monitoring is still needed (SGLT2 inhibitors may slightly increase warfarin concentration).

**Strengthen social support:**

- For patients living alone, cooperation with primary healthcare is needed:
  - Educate on dehydration signs (orthostatic hypotension, dark urine);
  - Provide a home scale (daily weight fluctuation >1.5 kg warrants alert);
  - Push medication reminders (via phone/smart devices).

**Not recommended alternatives:**

- GLP-1 receptor agonists: although they offer cardiovascular benefit (e.g., in the LEADER trial), they are inferior to SGLT2 inhibitors for reducing heart failure hospitalization and injectable administration may reduce adherence in individuals living alone.
- DPP-4 inhibitors: saxagliptin increased heart failure risk (SAVOR-TIMI 53), and others lack evidence of heart failure benefit.

**Conclusion:**
SGLT2 inhibitors represent the optimal choice for preventing heart failure recurrence, managing hyperglycemia, and protecting renal function in this patient. However, safe initiation requires prior volume optimization, coordinated monitoring with primary care, and robust patient education. If eGFR persistently declines below 45 mL/min or if recurrent urinary tract infections occur, consider switching to empagliflozin and reassess continuation.

**(3) Response (Gemini-2.5 Flash):**

As a clinical physician, I will provide a recommendation regarding the use of SGLT2 inhibitors for Mr. Wang based on his specific condition and the latest evidence-based medical literature, while clarifying the rationale and boundaries of this recommendation.

**Evidence-based recommendation for Mr. Wang:**

Dear Mr. Wang,
Based on your current condition—including a history of type 2 diabetes, a recent hospitalization due to acute decompensated heart failure with a diagnosis of heart failure with mildly reduced ejection fraction (HFmrEF, LVEF 45%), mild symptoms (NYHA Class II), and an eGFR of 62 mL/min/1.73m²—we strongly recommend the addition of a sodium-glucose cotransporter 2 inhibitor (SGLT2 inhibitor) to your current regimen, which includes a diuretic, ACEI, β-blocker, and warfarin.

**Rationale for recommendation:**

**Robust cardiovascular and renal protection evidence (especially for HF hospitalization and CV death):**

- **Benefit in HFmrEF patients:** Your LVEF of 45% falls under the category of HFmrEF. The recently published DELIVER trial (targeting HF patients with LVEF >40%, including HFmrEF and HFpEF) and EMPEROR-Preserved trial (focused on HFpEF with LVEF >40%, partly including HFmrEF) both showed that SGLT2 inhibitors (such as dapagliflozin or empagliflozin) significantly reduce the risk of the composite endpoint of cardiovascular death or heart failure hospitalization. These results directly support the use of SGLT2 inhibitors in patients like you.
- **Early initiation after acute HF hospitalization:** You were hospitalized for acute decompensated heart failure and are currently one week post-discharge. The SOLOIST-WHF study (using sotagliflozin, a dual SGLT1/SGLT2 inhibitor) demonstrated that initiating SGLT2 inhibitors during hospitalization or shortly after discharge (median of 3 days post-discharge) is safe and effective, significantly reducing the incidence of cardiovascular death and heart failure events. This suggests that early initiation in your case may offer additional benefits, stabilizing your condition and reducing the risk of rehospitalization.
- **Additional benefits for diabetic patients:** You have type 2 diabetes, and SGLT2 inhibitors were initially developed as glucose-lowering agents for diabetic patients. In trials like DAPA-HF and EMPEROR-Reduced, the heart failure benefits of SGLT2 inhibitors were consistent regardless of diabetes status. For diabetic patients, these drugs provide additional renal protection and glycemic control benefits, improving overall prognosis.

**Symptom improvement and quality of life:**
Clinical trials consistently show that SGLT2 inhibitors improve symptoms of heart failure (such as dyspnea and edema) and quality of life, aligning with your current treatment goal of improving NYHA Class II symptoms.

**Good safety profile:**
SGLT2 inhibitors have demonstrated favorable safety in large clinical trials. Common side effects include genital fungal infections, urinary tract infections, and volume depletion (especially during initiation). With your eGFR of 62 mL/min/1.73m², these drugs are safe to use and may slow renal function decline.

**Boundaries of clinical judgment:**

When adding an SGLT2 inhibitor, we need to consider the following points and conduct close monitoring:

- **Drug selection:** In China, dapagliflozin and empagliflozin are approved for heart failure treatment. Both have solid clinical evidence for use in HFrEF, HFpEF, and HFmrEF. The choice will depend on availability and patient-specific factors.
- **Starting dose and titration:** SGLT2 inhibitors are typically initiated at a fixed dose without the need for titration. However, during the initiation phase, careful monitoring of blood pressure and volume status is essential, especially since you were recently discharged and are still on a loop diuretic. The diuretic dose may need adjustment to prevent excessive diuresis leading to hypotension or acute kidney injury.
- **Renal function monitoring:**
  Although SGLT2 inhibitors protect renal function in the long term, a transient decrease in eGFR may occur in the first 2–4 weeks of treatment. This is usually reversible and within expected parameters. We will regularly monitor your renal function (serum creatinine and eGFR). For patients with eGFR <20–30 mL/min/1.73m², data on heart failure indications are limited, and use is either cautious or not recommended. Your current eGFR of 62 fully meets the criteria for use.
- **Dehydration and hypotension risk:**
  Due to their diuretic effect, combined with your current use of loop diuretics, there is a need to watch for dehydration and hypotension. We will guide you to monitor your blood pressure and symptoms closely and adjust your diuretic dosage as necessary.
- **Genitourinary infection risk:**
  Although relatively uncommon, SGLT2 inhibitors may increase the risk of urinary tract and genital fungal infections. We will inform you about these risks and provide guidance on personal hygiene.
- **Diabetic ketoacidosis (DKA) risk:**
  Although extremely rare, in specific conditions (e.g., fasting, major surgery, severe illness), patients taking SGLT2 inhibitors may develop euglycemic DKA. As a patient with type 2 diabetes, you should be informed and vigilant for related symptoms.
- **Patient education and follow-up:**
  Considering that you live alone and rely on primary care for follow-up, detailed patient education is essential. We will ensure you understand the drug's mechanism, administration, common side effects, and management strategies. We will also coordinate with your primary care center to ensure regular monitoring and follow-up.

**Conclusion:**
Taking into account your age, medical history, type of heart failure, recent hospitalization, and renal function, the addition of an SGLT2 inhibitor will significantly reduce your risk of future heart failure rehospitalization and cardiovascular death, improve your symptoms, and provide additional renal and glycemic control benefits. We will closely monitor your clinical response and any potential side effects and adjust the treatment plan as necessary to ensure you receive optimal therapy under the supervision of your local primary care facility.
